# Rapidly Progressive Subcutaneous Emphysema and Sequential Bilateral Pneumothoraces Following Pediatric Tracheocutaneous Fistula Closure: A Postoperative Air-Leak Syndrome Recognized in the Post-anesthesia Care Unit (PACU)

**DOI:** 10.7759/cureus.107440

**Published:** 2026-04-21

**Authors:** Dedric Bryant, Ajay Pius, Dominik Choromanski

**Affiliations:** 1 Department of Anesthesiology, McLaren Greater Lansing, Lansing, USA; 2 Department of Anesthesiology, Henry Ford Health System, Detroit, USA; 3 Department of Pediatric Anesthesiology, Children’s Hospital of Michigan, Detroit Medical Center, Detroit, USA

**Keywords:** airway surgery, direct laryngoscopy and bronchoscopy, pediatric anesthesia, pneumomediastinum, pneumothorax, postoperative complications, subcutaneous emphysema, tracheocutaneous fistula

## Abstract

Tracheocutaneous fistula (TCF) closure is commonly performed in pediatric patients following successful decannulation from long-term tracheostomy dependence. Although the procedure is generally considered safe, postoperative air-leak complications, including subcutaneous emphysema, pneumomediastinum, and pneumothorax, have been reported. Early recognition is critical because progression may lead to significant respiratory compromise. We report the case of a three-year-old child who developed rapidly progressive subcutaneous emphysema in the post-anesthesia care unit (PACU) following TCF closure after direct laryngoscopy and bronchoscopy. The patient subsequently developed massive subcutaneous emphysema that progressed to bilateral pneumothoraces requiring emergent chest tube placement and intensive care management. This case highlights the potential for rapid progression of postoperative air-leak syndromes following pediatric airway surgery and emphasizes the importance of early recognition by anesthesia providers during the immediate postoperative period. Awareness of the clinical presentation of subcutaneous emphysema and its potential progression to pneumomediastinum and pneumothorax is essential to prevent life-threatening complications.

## Introduction

Tracheostomy is frequently required in pediatric patients with congenital or acquired airway obstruction, prolonged ventilatory dependence, or complex airway disorders. Advances in neonatal and pediatric intensive care have significantly improved survival among children with chronic respiratory conditions, resulting in a growing population of pediatric patients living with long-term tracheostomies. Indications for tracheostomy in children include airway malformations, subglottic stenosis, tracheomalacia, neuromuscular disease, and chronic respiratory failure requiring prolonged mechanical ventilation [1].

As airway pathology improves and respiratory stability is achieved, decannulation (removal of the tracheostomy tube) may be considered. However, persistent tracheocutaneous fistula (TCF, defined as a persistent epithelialized tract connecting the tracheal lumen to the skin surface that fails to close following tracheostomy tube removal) develops in a substantial proportion of patients following decannulation as a result of epithelialization of the tract between the trachea and skin. Reported rates of persistent fistula formation vary widely in the literature, with incidences ranging from approximately 6% to 55%, particularly in patients with prolonged tracheostomy duration [1-3]. This persistence is attributed to chronic epithelialization of the tract and impaired spontaneous closure, especially in long-standing tracheostomies. Additional pediatric series have demonstrated that prolonged cannulation duration is one of the strongest predictors of persistent TCF formation following decannulation [2,3].

When spontaneous closure does not occur, surgical excision and primary closure of the fistula are commonly performed. Primary closure refers to direct surgical excision of the fistula tract followed by layered closure of the tracheal and cutaneous defects. The goals of surgical repair include restoration of normal airway anatomy, elimination of persistent air leakage, and prevention of recurrent respiratory infections associated with chronic fistula tracts [2-4]. Multiple surgical series have demonstrated high success rates with primary closure techniques, though complication rates vary depending on operative technique and perioperative airway management strategies [2,3]. Although TCF closure is generally considered a relatively minor surgical procedure, postoperative complications have been described.

Reported complications include wound infection, recurrence of the fistula, airway obstruction due to edema or hematoma formation, and air-leak syndromes such as subcutaneous emphysema, pneumomediastinum, and pneumothorax [2-5]. These air-leak complications, while uncommon, are consistently reported across pediatric surgical series and may present early in the postoperative period with rapid progression [2,3]. The incidence of postoperative subcutaneous emphysema following TCF closure varies across studies but has been reported to range from approximately 3% to 20%, depending on surgical technique and perioperative management strategies [5,6].

Air-leak complications following TCF closure occur when air escapes through an incompletely sealed tracheal closure or through microscopic defects created during surgical dissection of the fistula tract. Increased intrathoracic pressure generated by coughing, crying, or positive pressure ventilation may force air through these defects into surrounding tissues [4,7,8]. Positive airway pressure has specifically been implicated as a contributing factor to postoperative air leak following TCF closure, particularly in cases where tracheal closure is not fully sealed [8]. Once air enters the cervical fascial planes, it may rapidly track along tissue planes into the mediastinum and pleural spaces [4,9].

Subcutaneous emphysema is often the earliest manifestation of a postoperative air leak. Patients may develop progressive swelling of the face, neck, or chest with palpable crepitus resulting from air trapped within the soft tissues. While mild cases may resolve spontaneously, severe cases may progress to pneumomediastinum or pneumothorax and result in significant respiratory compromise.

In the perioperative setting, anesthesia providers frequently serve as the first clinicians to recognize these complications during emergence from anesthesia or in the post-anesthesia care unit (PACU). Early recognition and prompt evaluation are critical to prevent progression to life-threatening complications such as tension pneumothorax or mediastinal compression.

We present a case of rapidly progressive subcutaneous emphysema following pediatric TCF closure that ultimately progressed to sequential bilateral pneumothoraces requiring emergent chest tube placement and intensive care management.

## Case presentation

A three-year-old male (American Society of Anesthesiologists (ASA) III, BMI 17.4 kg/m², Z-score +1.78) with a history of severe congenital subglottic stenosis and prior tracheostomy dependence presented for direct laryngoscopy, bronchoscopy, excision of a vocal fold cyst, and surgical closure of a persistent TCF. The patient had previously undergone tracheostomy placement at one month of age for airway management related to severe subglottic stenosis and tracheomalacia.

At baseline, he was breathing comfortably on room air without recent respiratory symptoms or infectious concerns. Preoperatively, the patient was comfortable and well-appearing with oxygen saturation of 100% on room air and hemodynamic stability. He presented with a 3.5 mm uncuffed tracheostomy tube in situ and typically used a humidified tracheostomy collar at night.

The patient was brought to the operating room, where general anesthesia was induced with sevoflurane while maintaining spontaneous ventilation. A propofol infusion (300 mcg/kg/min) was initiated for direct laryngoscopy and bronchoscopy, supplemented with fentanyl (10 mcg) and a single bolus of dexmedetomidine (8 mcg) to maintain adequate depth of anesthesia and minimize airway reactivity. Neuromuscular blockade was not administered in order to preserve spontaneous respiration during airway evaluation.

Direct laryngoscopy and bronchoscopy were performed to evaluate the airway. Examination revealed normal supraglottic structures and a posterior true vocal fold cyst, which was successfully removed. Following completion of direct laryngoscopy and bronchoscopy, the otolaryngology team placed a 4.0 cuffed endotracheal tube to facilitate controlled ventilation for the remainder of the procedure. The TCF was then excised and surgically closed.

At the conclusion of the procedure, a 3.0 uncuffed tracheostomy tube was temporarily placed to maintain airway access during emergence from anesthesia. Emergence was performed under a deep plane of anesthesia to minimize coughing and agitation. The patient was transported to the PACU, breathing spontaneously with supplemental oxygen delivered via a face mask placed over the tracheostomy tube.

Following arrival in the PACU, when the patient started to wake up, the tracheostomy tube was removed by the otolaryngology team as planned. Shortly after decannulation, the patient developed respiratory distress characterized by tachypnea to approximately 40 breaths per minute, increased work of breathing, and coughing. During this period, oxygen saturation acutely declined to the 70s over two to three minutes, while blood pressure remained stable. The physician overseeing the PACU ordered albuterol and later racemic epinephrine due to concern for potential upper airway edema; however, symptoms persisted.

During further evaluation, rapidly increasing swelling of the cheeks, neck, and upper chest was observed. Palpation revealed extensive subcutaneous crepitus involving the cervical region, chest wall, and bilateral upper extremities. These findings raised immediate concern for a significant postoperative air leak.

**Figure 1:**
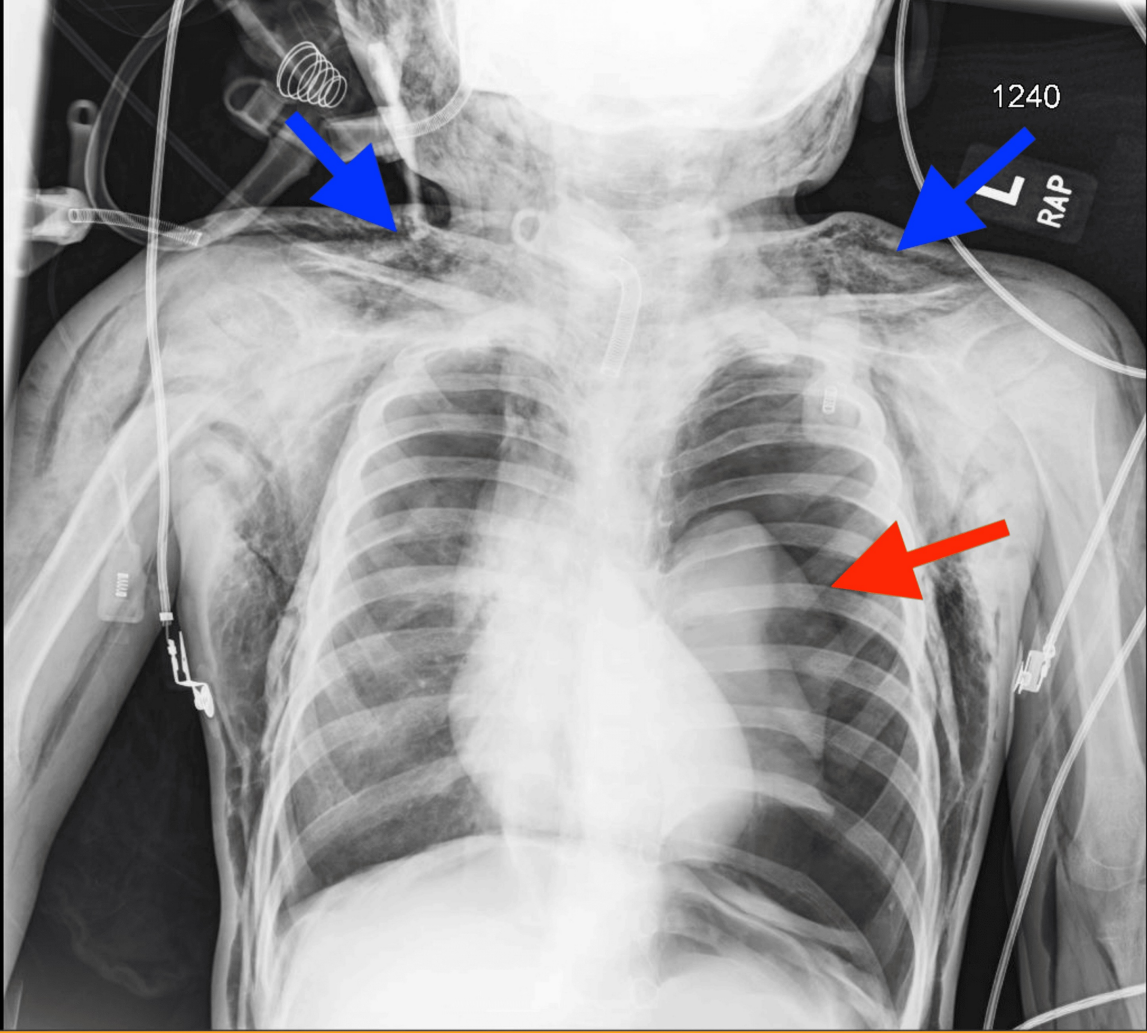
Initial chest radiograph findings Demonstrating a left-sided pneumothorax (red arrow) with extensive cervical and thoracic subcutaneous emphysema (blue arrows).

The otolaryngology team was urgently called to the bedside, and the tracheostomy tube was reinserted in an attempt to relieve airway pressure and facilitate ventilation. A chest radiograph obtained shortly thereafter demonstrated a large left-sided pneumothorax with near-complete collapse of the left lung, along with extensive subcutaneous emphysema involving the neck, chest wall, and upper extremities (Figure 1).

The patient was re-sedated with a propofol bolus (20 mg), administered fentanyl (10 mcg), and paralyzed with rocuronium (15 mg) to facilitate the placement of the left-sided chest tube. He was subsequently placed on mechanical ventilation using a pressure-controlled mode, with a strategy focused on minimizing airway pressures while maintaining adequate oxygenation and ventilation.

A left-sided chest tube was placed at the bedside with immediate release of air and subsequent re-expansion of the lung on repeat imaging. The patient was transferred to the pediatric intensive care unit for further monitoring and management.

**Figure 2:**
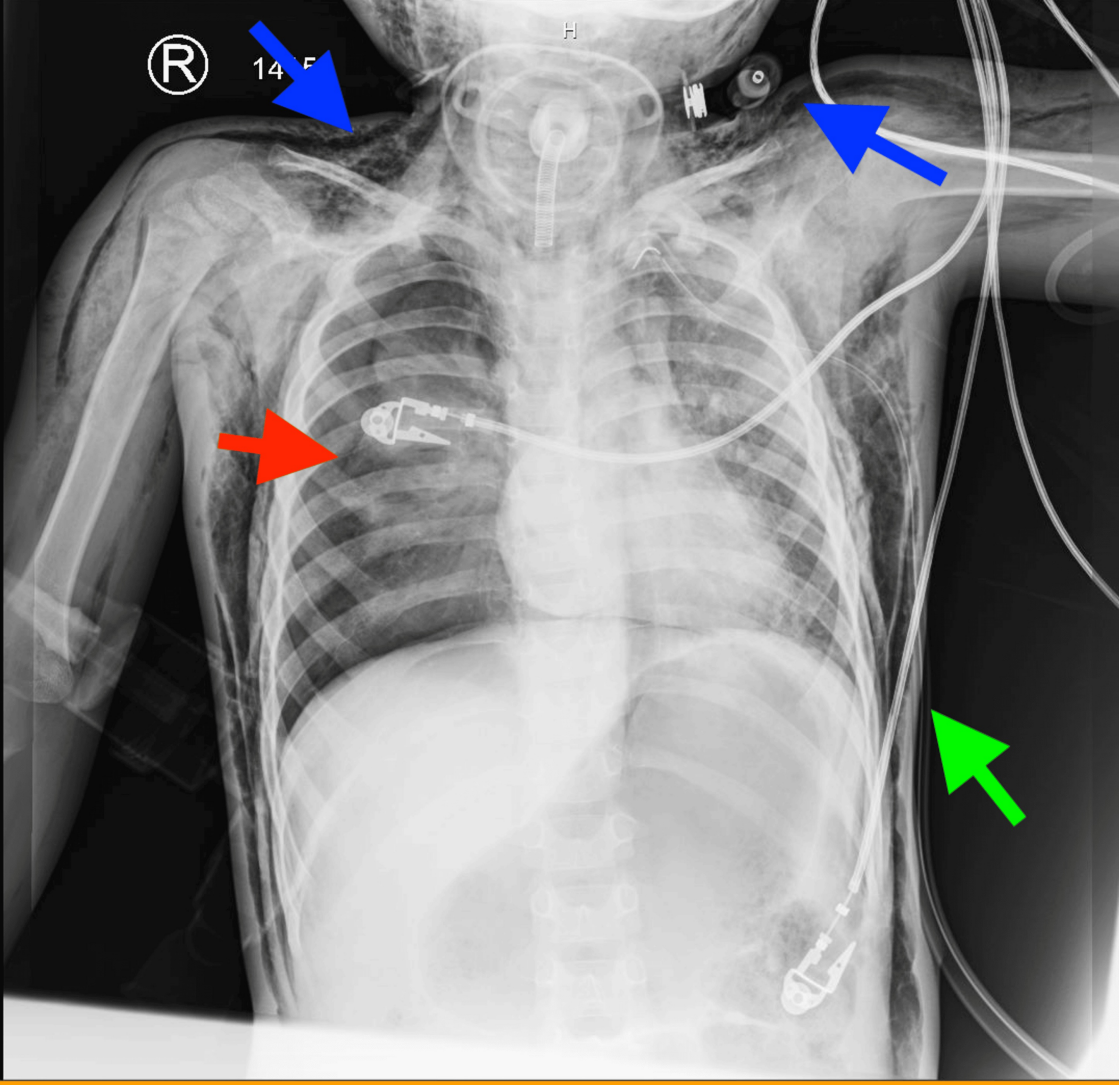
Follow-up chest radiograph findings Demonstrating re-expansion of the left lung with a left-sided chest tube in place (green arrow) and interval development of a right-sided pneumothorax (red arrow). Persistent subcutaneous emphysema is again noted (blue arrows).

Upon arrival in the pediatric intensive care unit, the patient initially demonstrated tachypnea but subsequently developed worsening respiratory distress. Repeat chest imaging approximately one hour later revealed interval development of a right-sided pneumothorax in addition to persistent pneumomediastinum and extensive subcutaneous emphysema (Figure 2).

**Figure 3:**
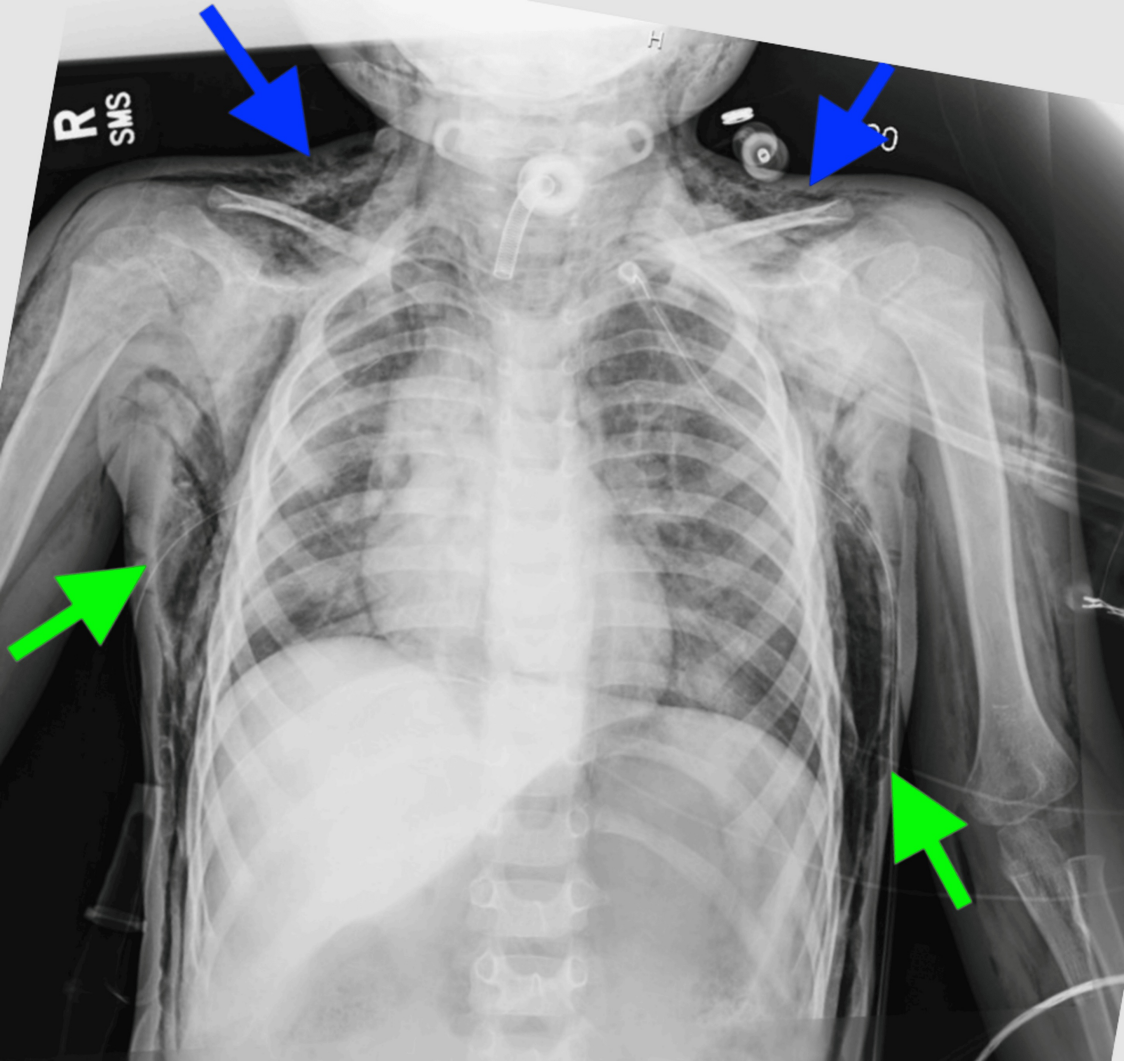
Chest radiograph findings Demonstrating bilateral chest tubes (green arrows) with re-expansion of both lungs. Residual subcutaneous emphysema persists (blue arrows).

A right-sided chest tube was subsequently placed with continued ventilatory support. Subsequent imaging demonstrated re-expansion of both lungs with bilateral chest tubes in place (Figure 3).

The patient remained under close monitoring in the pediatric intensive care unit with supportive care. Sedation was maintained with fentanyl and dexmedetomidine infusions in the ICU setting. Chest tubes were successfully removed after two days, and subcutaneous emphysema showed significant resolution within 24 hours. The patient ultimately demonstrated clinical improvement without further complications.

## Discussion

Respiratory distress occurring shortly after airway surgery presents a diagnostic challenge in the PACU. In pediatric patients, the differential diagnosis for acute postoperative respiratory compromise includes airway edema, laryngospasm, bronchospasm, residual anesthetic effects, hemorrhage causing airway obstruction, and allergic or anaphylactic reactions to perioperative medications.

In this case, the initial differential diagnosis included airway edema and possible anaphylaxis due to the presence of respiratory distress shortly after surgery. Acute postoperative bleeding was also considered, given the recent airway procedure. However, the rapid development of facial swelling accompanied by palpable crepitus strongly suggested the presence of subcutaneous emphysema and shifted concern toward an underlying air-leak syndrome.

Air-leak complications following TCF closure are uncommon but well-documented in the pediatric airway literature [5]. These complications occur when air escapes through defects in the tracheal closure or through disruptions created during surgical dissection of the fistula tract. Elevated intrathoracic pressures generated by coughing, crying, agitation, or positive pressure ventilation may force air through these defects into surrounding tissues [4,7,8]. Prior studies have demonstrated that positive pressure ventilation is associated with increased risk of complications following pediatric TCF repair, likely due to increased trans-tracheal pressure gradients promoting air dissection into surrounding tissue planes [8].

Once air enters the cervical fascial planes, it may rapidly dissect along tissue planes into the mediastinum, resulting in pneumomediastinum and extensive subcutaneous emphysema [9,10]. The progression of air along fascial and bronchovascular planes is consistent with mechanisms described in spontaneous pneumomediastinum, where alveolar rupture leads to air tracking centrally into the mediastinum and subsequently into subcutaneous tissues or pleural spaces [10]. Progression to pneumothorax may occur when increasing mediastinal pressure leads to rupture of the mediastinal pleura, allowing air to enter the pleural space. Algorithms for spontaneous pneumomediastinum emphasize that worsening respiratory distress or radiographic progression should prompt evaluation for secondary pneumothorax, as demonstrated in this case [10].

The underlying pathophysiology of these air-leak syndromes has been described extensively in the literature. Maunder and colleagues characterized the spread of air within mediastinal connective tissue planes following disruption of airway integrity [9]. Additionally, the Macklin effect describes the movement of air from ruptured alveoli along bronchovascular sheaths into the mediastinum, which may subsequently extend into cervical soft tissues or the pleural space [11].

In the present case, several perioperative factors likely contributed to the development and progression of the observed air-leak syndrome. Surgical disruption of the TCF tract creates a potential pathway for air escape, particularly if the tracheal closure is not completely sealed. Episodes of coughing and agitation during emergence, along with transient use of positive pressure ventilation, may have increased intrathoracic pressures and facilitated air dissection into surrounding tissue planes. These risk factors are particularly relevant in pediatric patients, who can generate high intrathoracic pressures during distress and have relatively compliant mediastinal tissues that allow rapid propagation of air. Recognition of these factors underscores important perioperative strategies to mitigate risk, including minimizing positive pressure ventilation when feasible, ensuring meticulous surgical closure, and employing anesthetic techniques that promote a smooth emergence to reduce coughing and agitation.

Pediatric patients may be particularly vulnerable to the rapid progression of these air-leak syndromes. Children can generate substantial intrathoracic pressures during crying or agitation in the immediate postoperative period, which may force air through a partially sealed tracheal closure. Furthermore, the compliant nature of pediatric mediastinal tissues may allow air to dissect rapidly through fascial planes.

Recognition of subcutaneous emphysema in the PACU is therefore critical. Clinically, subcutaneous emphysema presents as progressive swelling of the face, neck, or chest accompanied by palpable crepitus. This finding should immediately raise suspicion for an underlying air-leak process and prompt urgent imaging and multidisciplinary evaluation.

In the present case, recognition of rapidly progressive subcutaneous emphysema prompted urgent evaluation that led to identification of a large pneumothorax requiring immediate decompression. Despite initial treatment, the patient subsequently developed a contralateral pneumothorax, illustrating the potential for ongoing air dissection and delayed progression of air-leak syndromes even after initial intervention.

Although postoperative air-leak complications following tracheocutaneous fistula closure are described in the otolaryngology literature, reports emphasizing early recognition in the PACU from the perspective of anesthesia providers remain limited. Prior cases of severe postoperative subcutaneous emphysema with associated respiratory compromise underscore the potential for rapid clinical deterioration in the immediate postoperative period [12]. However, much of the existing literature focuses primarily on surgical technique and operative outcomes, with comparatively less attention given to the diagnostic challenges encountered during early recovery. Given that anesthesia clinicians are often the first to evaluate patients during emergence and in the PACU, timely recognition of atypical findings such as rapidly progressive subcutaneous emphysema is essential to facilitate prompt intervention and prevent progression to life-threatening complications.

## Conclusions

Subcutaneous emphysema following TCF closure is an uncommon but potentially serious complication that may indicate the presence of pneumomediastinum or pneumothorax. This case highlights how air-leak syndromes can develop and progress rapidly in the immediate postoperative period, particularly in the setting of incomplete tracheal closure combined with increased intrathoracic pressures from coughing, agitation, or positive pressure ventilation. These findings underscore the importance of perioperative strategies aimed at minimizing positive pressure ventilation when feasible and promoting a smooth, controlled emergence to reduce abrupt increases in intrathoracic pressure.

Given the potential for rapid deterioration, early recognition of clinical signs such as facial or cervical swelling with palpable crepitus should prompt urgent evaluation and intervention. Vigilance in the PACU, along with timely imaging and coordinated multidisciplinary management, is critical to prevent progression to life-threatening complications, as even transient delays in recognition may result in rapid evolution to bilateral pneumothoraces and respiratory compromise.
